# Correction: Ebrahim et al. The Prevalence of Falls Among Older Adults Living in Long-Term Care Facilities in the City of Cape Town. *Int. J. Environ. Res. Public Health* 2025, *22*, 432

**DOI:** 10.3390/ijerph22050798

**Published:** 2025-05-20

**Authors:** Nabilah Ebrahim, Jaron Ras, Rucia November, Lloyd Leach

**Affiliations:** Department of Sport, Recreation and Exercise Science, Faculty of Community and Health Sciences, University of the Western Cape, Robert Sobukwe Rd, Bellville, Cape Town 7535, South Africa; rasj@cput.ac.za (J.R.); novemberru@cput.ac.za (R.N.); lleach@uwc.ac.za (L.L.)

## Addition of Two Authors

Jaron Ras and Rucia November were not included as authors in the original publication [1]. The updated authorship now appropriately reflects their contributions. Jaron Ras was involved in conceptualization, methodology, validation, writing—original draft preparation, visualization, and supervision. Rucia November contributed to conceptualization, methodology, software development, validation, formal analysis, data curation, writing—review and editing, visualization, supervision, and project administration. The affiliations and author contributions statement have been revised accordingly to accurately reflect these updates.

The authors state that the scientific conclusions are unaffected. This correction was approved by the Academic Editor. The original publication has also been updated.
